# Optimizing hemodialysis management using bioelectrical impedance analysis: emphasizing the significance of the phase angle

**DOI:** 10.1080/0886022X.2025.2567037

**Published:** 2025-10-05

**Authors:** Jungho Shin, Hongtae Kim, Hae Eun Jeon, Soie Kwon, Jin Ho Hwang, Hyung-Eun Son, Semin Cho, Jeong Min Cho, Su Hyun Kim

**Affiliations:** ^a^ Department of Internal Medicine, Chung-Ang University Hospital, Seoul, South Korea; ^b^ Department of Internal Medicine, Chung-Ang University Gwangmyeong Hospital, Gwangmyeong, South Korea

**Keywords:** Phase angle, bioimpedance, hemodialysis, sarcopenia

## Abstract

Patients undergoing hemodialysis (HD) often experience complications, such as severe malnutrition, muscle wasting, and fluid overload, which significantly affect survival rates and quality of life. Accurate assessment and management of nutritional status and fluid retention are essential for improving patient outcomes. Bioelectrical impedance analysis (BIA), a noninvasive and practical tool, is increasingly used to assess body composition and fluid distribution in patients undergoing HD. The phase angle (PhA), a BIA parameter, reflects cellular integrity and serves as a reliable indicator of nutritional status and clinical prognosis. This review discusses the clinical utility of BIA in assessing nutritional status, fluid retention, and body composition in patients undergoing maintenance HD, with a particular focus on the PhA. The role of the PhA in predicting survival, frailty, sarcopenia, and erythropoiesis-stimulating agent resistance was explored. The application of personalized dry weight management guided by BIA has been proposed to optimize the care of patients undergoing HD. Despite limitations, including inter-device variability, lack of standardized cutoff values, and limited evidence, BIA represents a promising tool that may enhance clinical outcomes if integrated into routine clinical practice.

## Introduction

Chronic kidney disease, which affects approximately 10% of the global population, represents a significant public health challenge [1]. Hemodialysis (HD) is increasingly used to treat patients with chronic kidney disease, with the 2020 United States Renal Data System reporting that 492,987 patients were undergoing HD in the United States [2]. In Korea, data from the Korean Renal Data System indicate that 83.6% of all patients undergoing dialysis in 2019 received HD [3].

Patients undergoing maintenance HD often experience complications related to uremia, such as malnutrition, muscle wasting, and fluid overload. These complications significantly compromise patient survival and quality of life [4,5]. Fluid overload, which results from excess fluid retention and is a critical form of body composition imbalance, has been linked to significantly increased mortality in patients undergoing HD. In a multicenter study of 392 patients undergoing HD, Zoccali et al. reported a higher risk of death (hazard ratio [HR] 4.20; 95% confidence interval [CI] 2.45–7.23) in patients with very severe pulmonary congestion than in those with mild or no congestion (as assessed by B-lines on lung ultrasound) [6]. Therefore, regular and objective assessments of nutritional status, fluid retention, and body composition in patients undergoing HD is essential to improve survival rates and quality of life.

Bioelectrical impedance analysis (BIA) is a noninvasive and relatively low-cost tool that can effectively estimate the volume status, body composition, and nutritional status of patients undergoing maintenance HD. One key parameter, the phase angle (PhA), reflects cell membrane integrity and cellular health; lower PhA values are associated with poorer nutritional status and worse clinical outcomes [7].

This review aimed to explore the methodologies used to evaluate the nutritional status, fluid retention, and body composition of patients on maintenance HD using BIA, with particular emphasis on the clinical utility of the PhA. We discuss current clinical applications, limitations, and future directions for the integration of these techniques into routine clinical practice.

## Principles and techniques of BIA

**Figure 1.**
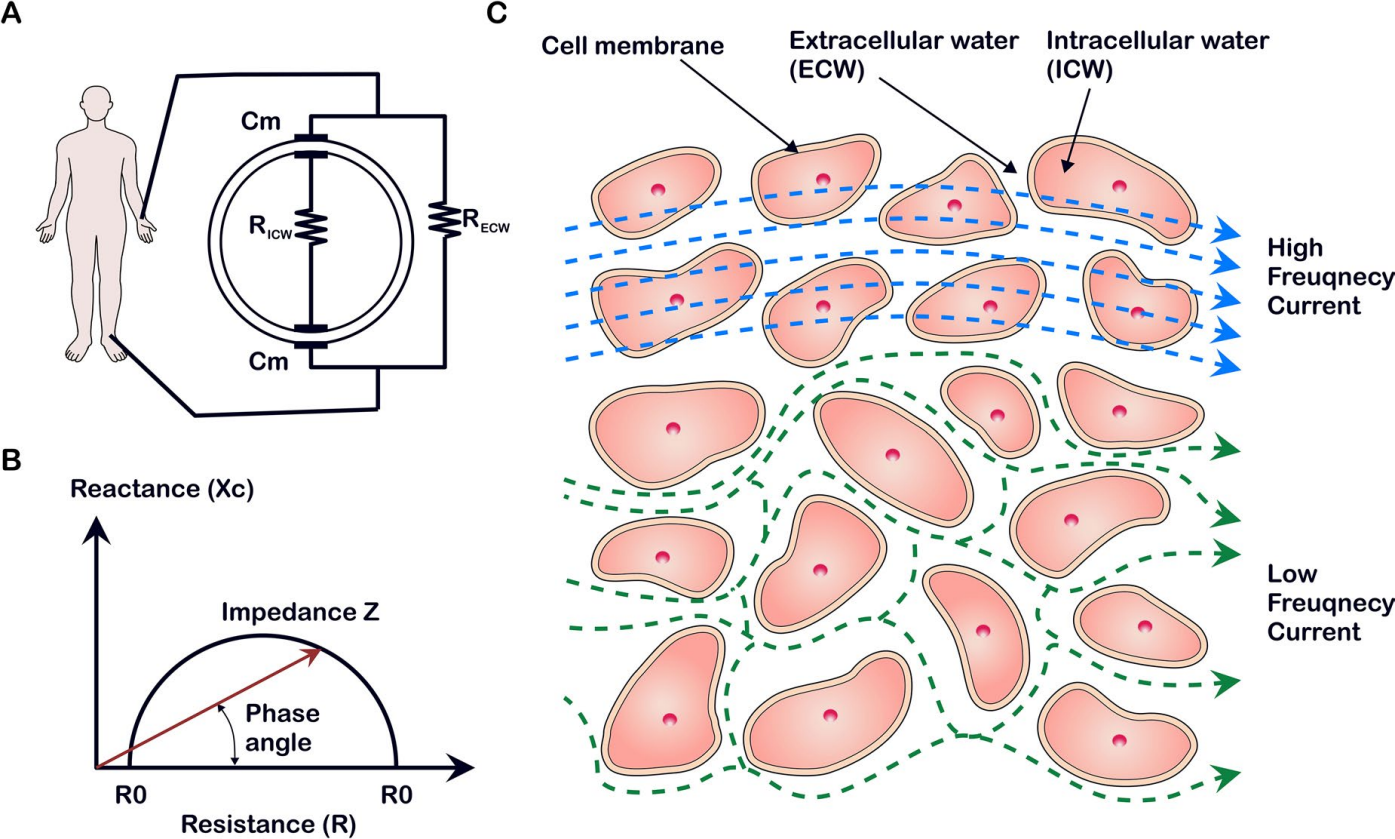
Bioelectrical impedance analysis. (A) Principle of bioelectrical impedance analysis, considering the human body as a conductor with complex electrical properties. (B) The resistance (R) and reactance (Xc) are used to determine the impedance (Z) and phase angle. (C) Schematic representation of low-frequency current passing through extracellular water and high-frequency current passing through both extracellular and intracellular water.

BIA is based on the principle that the human body can be regarded as an electrical conductor, with its various tissues forming a composite circuit (Figure 1(A)). By applying an electrical current and measuring the impedance (Z) from the resistance (R) and reactance (Xc), fluid distribution and body composition can be estimated (Figure 1(B)). The PhA, calculated as the arctangent of Xc divided by R (PhA = arctan(Xc/R) × 180°/π), represents the phase shift between applied voltage and measured current and reflects cellular integrity. Table 1 shows parameters derived from BIA and their clinical meanings.

**Table 1.** Primary Bioelectrical impedance analysis parameters and their clinical significance.

Parameter	Definition/Formula	Unit	Clinical Significance	Clinical Use example
R	Opposition to current flow through body	Ω	- Lower in tissues with higher muscle/fluid content - Higher in adipose (fat) tissues	
Xc	“Capacitive” component arising from cell membranes	Ω	- Reflects cell membrane integrity and mass - Increases with cellular content	
Z	Vector sum of R and Xc	Ω	- Represents the total electrical resistance of the body	
Phase angle	Phase shift between R and Xc	°	- Indicates cell membrane health and nutritional status - Lower values suggest poor prognosis	Risk stratification and monitoring; cutoff values are device‑ and population‑specific
ICW	Water content within cells	L	- Reflects cellular function and hydration status	Use ECW/TBW ratio to estimate fluid overload and to adjust dry weight targets
ECW	Water content outside cells	L	- Assesses edema, fluid overload, and dehydration	
TBW	ICW + ECW	L	- Assesses overall fluid status; crucial in patients undergoing dialysis	
Lean body mass	Total mass of muscle and other nonfat tissues	kg	- Evaluates muscle status; useful for detecting sarcopenia and assessing nutritional status	Sarcopenia/nutrition monitoring
Fat mass	Total amount of adipose tissue (subcutaneous and visceral)	kg	- Assesses overweight/obesity and imbalances in body composition	Nutrition and body composition monitoring

ECW, extracellular water; ICW, intracellular water; TBW, total body water; R, resistance; Xc, reactance; Z, impedance.

BIA is classified as single-frequency BIA (SF-BIA), multiple-frequency BIA (MF-BIA), and bioelectrical impedance spectroscopy (BIS) according to the frequency range applied [8–10]. SF-BIA typically employs a 50 kHz current and estimates total body water (TBW) using population-based regression equations, whereas MF-BIA measures impedance at several discrete frequencies (commonly ranging from 5 to 1,000 kHz) and relies on empirical prediction formulas. In contrast, BIS acquires impedance over the entire frequency spectrum and applies the Cole–Cole model to extrapolate the resistance values at zero and infinite frequencies, thereby deriving extracellular water (ECW) and intracellular water (ICW) in a model-based manner, fundamentally different to those of SF-BIA and MF-BIA.

The adoption of 50 kHz as the standard for SF-BIA stems from early studies showing that this frequency demonstrated optimal correlation with TBW in healthy adults [11]. However, this uniform 50 kHz application is a fundamental limitation of SF-BIA, as it does not account for individual body composition characteristics or fluid distribution abnormalities. In particular, hypervolemia or hypovolemia alters the electrical properties of cell membranes, and impedance at 50 kHz may not accurately reflect actual fluid volumes [12].

Most studies cited in this review have used the 50 kHz PhA, consistent with conventional SF-BIA. Traditional BIA equations mainly rely on resistance because height^2^/R closely reflects body water, whereas the PhA shows only a weak association with overall body composition [11]. Nevertheless, the 50 kHz PhA retains independent value in nutritional and prognostic assessment and is therefore widely employed in clinical practice.

The Kidney Disease Outcomes Quality Initiative has recommended performing BIA at least 30 min after HD cessation because of the rapid fluid redistribution that occurs during the first 20–30 min post-dialysis [13]. This recommendation has been supported by a systematic study that continuously measured BIA parameters in 27 patients undergoing HD, in which the R, Xc, and PhA sharply increased to peak values immediately after dialysis and then remained stable for 15–120 min, indicating the period most accurately reflecting volume status [14]. Therefore, for patients undergoing three HD session per week, BIA measurements obtained during the mid-week session between 30 and 120 min post-dialysis, with the patient resting in the supine position for 5–10 min after removing metal accessories and avoiding limb contact, provide the most reliable assessment of fluid status.

## Clinical application in hemodialysis

### Fluid management and dry weight assessment

Chronic fluid overload is experienced by 40–50% of patients undergoing HD and is closely associated with hypertension, cardiovascular disease, and increased mortality [15–17]. An analysis by Zoccali et al. of 39,566 patients undergoing HD in 26 countries demonstrated that baseline fluid overload increased mortality risk by 26%; accumulated fluid overload exerted a greater influence on mortality risk [17]. Conversely, fluid depletion can lead to intradialytic hypotension, fatigue, and an increased mortality risk [18,19].

Achieving an optimal fluid balance is central to the management of patients undergoing HD, and setting an appropriate dry weight is crucial. Dry weight was initially defined as the post-dialysis weight at which blood pressure remained normal without antihypertensive medication, but has since been redefined as the lowest tolerable weight without significant hypovolemic or hypervolemic symptoms [20]. Nonetheless, determining dry weight remains challenging, as illustrated by studies reporting that 40.3% of patients undergoing HD exhibited fluid overload and 35.1% exhibited fluid depletion [21].

Various tools, including BIA, blood volume monitoring, B-lines on lung ultrasound, inferior vena cava diameter as determined by echocardiography, clinical assessments, and biomarkers such as brain natriuretic peptide, have been used to evaluate fluid status [22]. However, standardized diagnostic tools remain lacking. Other than BIA, most tools detect the presence of fluid overload or depletion rather than quantifying its degree, making it difficult for clinicians to determine patient-specific dry weight goals. BIA is considered a promising tool for fluid status evaluation. Moreover, BIS can help quantify reductions in extracellular volume, as demonstrated in a study by Kraemer et al. [23], which investigated the detection limits of several methods of fluid status assessment in patients undergoing dialysis. In this study, BIS demonstrated a detection limit of 0.87 ± 0.64 L, significantly lower than those of inferior vena cava diameter (1.74 ± 1.56 L) and relative blood volume monitoring (2.3 ± 1.0 L). However, because BIS evaluates whole-body hydration rather than directly assessing intravascular volume, caution is required in its interpretation, and it should be used to support dry weight determination in conjunction with clinical assessments, functional evaluations, and patient-reported symptoms.

Studies have shown that BIA-guided dry weight control significantly improves left ventricular mass index, blood pressure, survival, arterial stiffness, and relative fluid overload in patients undergoing maintenance HD [24–27]. Furthermore, BIA-guided fluid management has been reported to be economically beneficial owing to the reduced hospitalization rate. Stigger et al. [28] found that the hospitalization rate decreased from 3.1 per person-year in the usual care group to 2.1 per person-year in the BIA group (incidence rate ratio 1.48, *p* = 0.0001). The results of the recent BioImpedance Spectroscopy to maintain Renal Output randomized controlled trial also supported these findings, showing that BIA-guided fluid management was associated with an average cost per patient £382 lower than that of the control group, and 0.043 more quality-adjusted life-years, representing a 76% probability of being cost-effective at the £20,000/quality-adjusted life year threshold [29]. BIA is now increasingly used to manage fluid balance in patients with kidney diseases [30,31]. The detection of fluid overload by BIA has been shown to correlate with elevated blood pressure, increased left ventricular mass index, and a higher mortality risk [32,33], as well as with N-terminal pro B-type natriuretic peptide levels and echocardiographic findings [33].

**Figure 2.**
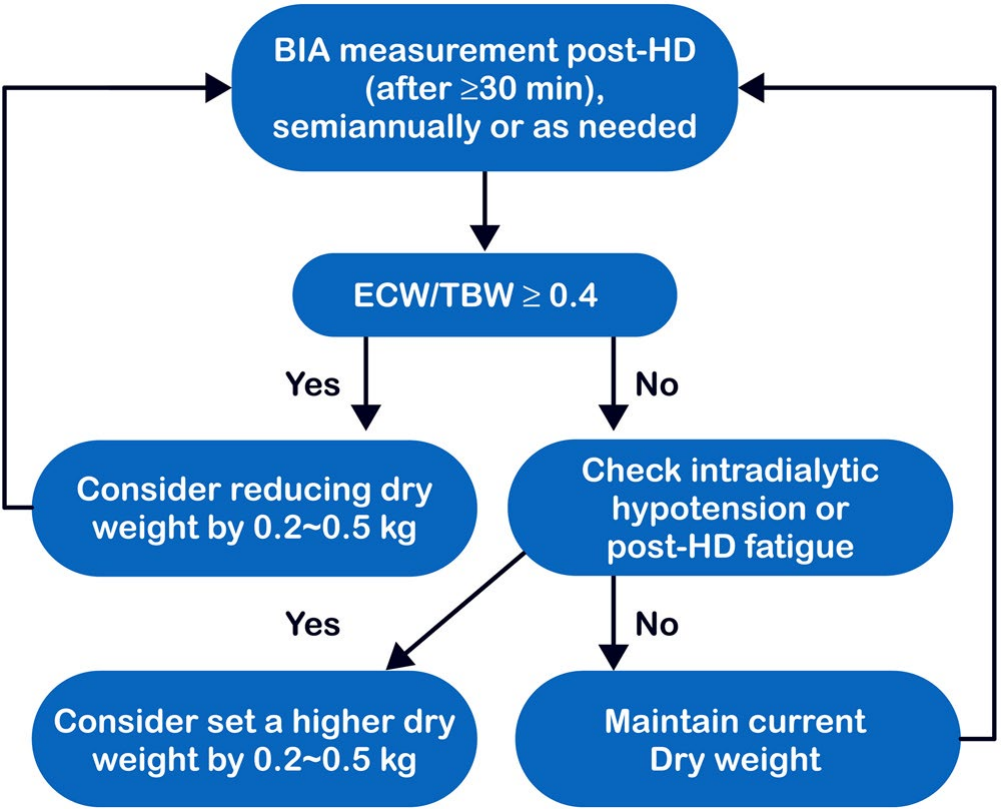
BIA-guided dry weight adjustment algorithm. BIA is performed ≥30 min post-HD, semiannually or as clinically indicated. If ECW/TBW ≥0.40, consider reducing dry weight by 0.2–0.5 kg; otherwise maintain current dry weight and continue routine monitoring. Thresholds and increments should be adapted to device- and population-specific references, as well as patient symptoms such as intradialytic hypotension and cramps. ECW, extracellular water; HD, hemodialysis; TBW, total body water.

Devices such as the Body Composition Monitor (Fresenius Medical Care, Bad Homburg, Germany) and InBody S10 (InBody, Seoul, South Korea) are increasingly used in South Korean dialysis units. In our center at Chung-Ang University Hospital, the InBody S10 is used to perform twice-yearly segmental MF-BIA in all patients undergoing HD as part of routing monitoring. Based on our clinical experience, we have developed a practical algorithm for BIA-guided dry weight adjustment (Figure 2). BIA measurements are performed (≥30 min post-hemodialysis, semiannually or as needed. When the ECW/TBW ratio is ≥0.4, reflecting fluid overload, we consider reducing dry weight by 0.2 to 0.5 kg. Patients with an ECW/TBW <0.4 are assessed for intradialytic hypotension or other complaint-related hypovolemia; if present, we consider increasing dry weight by 0.2 to 0.5 kg, or otherwise maintain the current dry weight. However, the ECW/TBW ratio should not be an absolute determinant, and the attending physician should make decisions considering the clinical status of the patient. This systematic approach helps optimize fluid balance while minimizing hemodynamic complications.

### Body composition analysis using BIA

Body composition analysis is critical for evaluating muscle and fat mass, which indicate nutritional status. This enables the early detection of conditions such as malnutrition and sarcopenia in patients undergoing dialysis, ultimately improving outcomes. BIA therefore represents a noninvasive and practical method of effectively identifying sarcopenia and malnutrition.

Dual-energy X-ray absorptiometry (DEXA) has also been used to assess body composition in patients undergoing HD [34,35]. A study of 53 stable adult patients undergoing HD found that MF-BIA and DEXA results were highly correlated in terms of lean body mass (*r* = 0.92) and fat mass (*r* = 0.93), although bone mineral content tended to be slightly overestimated using MF-BIA (*r* = 0.77) [36]. This overestimation may be attributed to the gap between the equation-based derivative from a general healthy population and the actual bone mineral content, particularly as influenced by the extraosseous calcification observed in chronic kidney disease [37].

**Figure 3.**
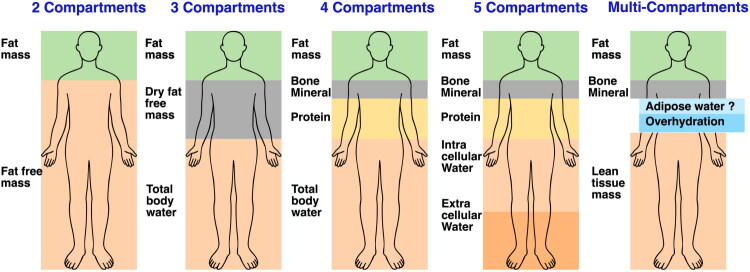
Overview of body composition models. The two-compartment model simply divides the body mass into fat and fat-free mass. A typical three-compartment model may separate total body water from fat-free mass or alternatively separate bone mineral as a distinct component. In the four-compartment model, fat mass, protein, total body water, and bone mineral are measured individually. The five-compartment model further subdivides total body water into intracellular and extracellular compartments. Multi-compartment models can be expanded further depending on research or clinical needs.

Body composition models can be refined from simple two-compartment models (fat and fat-free mass) to three-, four-, or even five-compartment models that include TBW, protein, and bone minerals [8,38–40] (Figure 3). Fluid imbalance can result in errors in conventional two-compartment BIA, as fluid is included in the fat-free mass compartment, potentially distorting nutritional status assessments. This issue can be addressed using multi-compartment BIA that divides fat-free mass into lean tissue mass and overhydration [40], thereby improving the precision and clinical utility of body composition assessment in patients undergoing maintenance HD [38,39].

Body composition analysis may increase understanding of the differences in intradialytic blood pressure measurements between the sexes. Females typically have lower muscle mass and higher body fat percentages than males, leading to lower TBW levels than those of males with the same body weight [41]. As muscle is crucial for maintaining fluid balance, women may exhibit more pronounced intravascular volume depletion during ultrafiltration, which could contribute to an increased risk of intradialytic hypotension. Indeed, a large-scale study of 16,959 patients reported that the incidence of hypotension was significantly higher in female patients undergoing dialysis than in males (12.1% vs. 10.1%, *p* < 0.001), with a 1.39-fold higher risk in females (95% CI 1.27–1.52) [42]. Another study of 112 dialysis patients found that systolic blood pressure fluctuation during HD was greater in female patients than in males (27.5 ± 12.9 vs. 21.9 ± 11.1 mmHg, *p* = 0.02) [43]. These findings may be explained, at least in part, by sex-based differences in body composition.

Sarcopenia is common in patients undergoing HD and is associated with increased morbidity and mortality [44,45]. In patients with chronic kidney disease, particularly those receiving maintenance HD, muscle mass is lost due to increased protein catabolism and reduced protein synthesis, leading to a negative protein balance [46].

Although interest in sarcopenia is increasing, the absence of a universally agreed upon definition limits its clinical application [47]. The 2019 Asian Working Group for Sarcopenia guidelines established diagnostic criteria for sarcopenia, integrating muscle strength as assessed by the handgrip strength test, physical function as assessed by the 6-m walk test or the Short Physical Performance Battery, and appendicular skeletal muscle mass as measured using DEXA or BIA. An appendicular skeletal muscle mass of <7.0 kg/m^2^ for men and <5.7 kg/m^2^ for women, combined with reduced muscle strength or function, indicates sarcopenia [48]. BIA is preferred over costlier and less accessible imaging modalities, such as computed tomography and magnetic resonance imaging, in routine clinical practice, and has been effectively used in recent studies of patients with chronic kidney disease [49].

### Nutritional status assessment using BIA

The Global Leadership Initiative on Malnutrition (GLIM) defines malnutrition based on phenotypic criteria, such as weight loss, low body mass index, and reduced muscle mass, and etiologic criteria, such as reduced food intake or assimilation and inflammation [50]. BIA can quickly estimate the fat-free mass index and other parameters, making it a suitable tool for patients undergoing dialysis who have limited access to DEXA. Studies have shown that patients undergoing HD who meet the GLIM criteria for malnutrition have higher mortality and hospitalization rates [51], underscoring the importance of early detection and intervention. Malnutrition, cachexia, and sarcopenia often overlap in patients undergoing HD [52].

### Other applications of BIA

Erythropoiesis-stimulating agent (ESA) resistance is common in patients with chronic kidney disease due to complex factors, including iron deficiency, uremic toxins, elevated parathyroid hormone levels, comorbidities, inflammation, and malnutrition [53]. Chronic inflammation negatively affects erythropoiesis, whereas malnutrition hinders adequate red blood cell production. These factors increase the risks of cardiovascular events and mortality.

**Figure 4.**
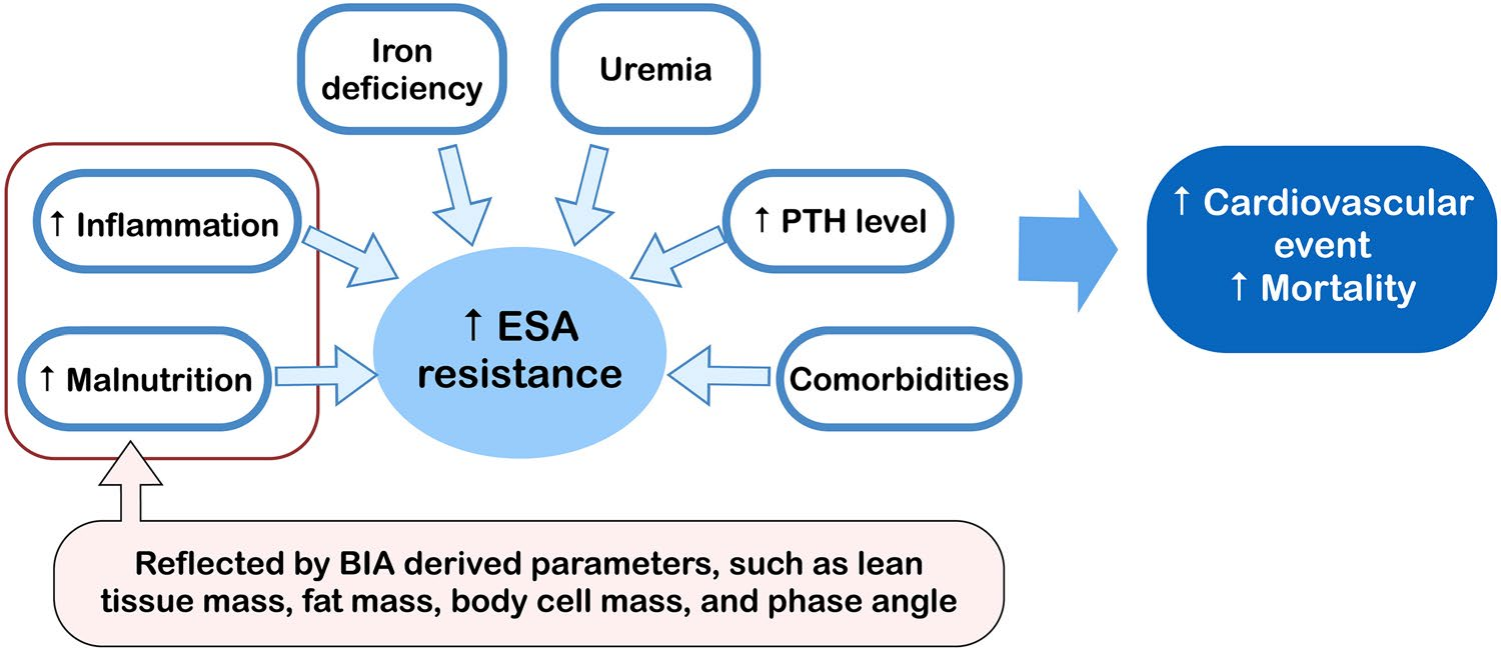
Determinants of ESA resistance and its impact. Inflammation, malnutrition, iron deficiency, uremia, elevated PTH, and comorbidities all increase ESA resistance and are associated with a higher risk of cardiovascular events and mortality. Among these factors, inflammation and malnutrition are assessable with BIA-derived parameters such as lean tissue mass, fat mass, body cell mass, and the phase angle. BIA, bioelectrical impedance analysis; ESA, erythropoiesis-stimulating agent; PTH, parathyroid hormone.

Inflammation and malnutrition are closely related to changes in body composition, and a reduction in fat and muscle mass can exacerbate ESA resistance [54]. These reductions have been independently associated with an elevated erythropoietin resistance index in patients undergoing long-term HD, which indicates reduced ESA sensitivity and is linked to higher risks of all-cause mortality, cardiovascular events, and infection [55]. The same study also reported a strong association between an elevated erythropoietin resistance index and a low PhA. Because BIA can assess malnutrition and inflammation (Figure 4), with muscle and fat mass reflecting nutritional status, and the PhA reflecting both nutritional and inflammatory status, serial BIA-assessed body composition analysis may quantitatively predict ESA responsiveness. Furthermore, targeted interventions, such as tailored nutritional supplementation and exercise programs, may help ameliorate ESA resistance and improve clinical outcomes.

## Phase angle a prognostic marker

**Figure 5.**
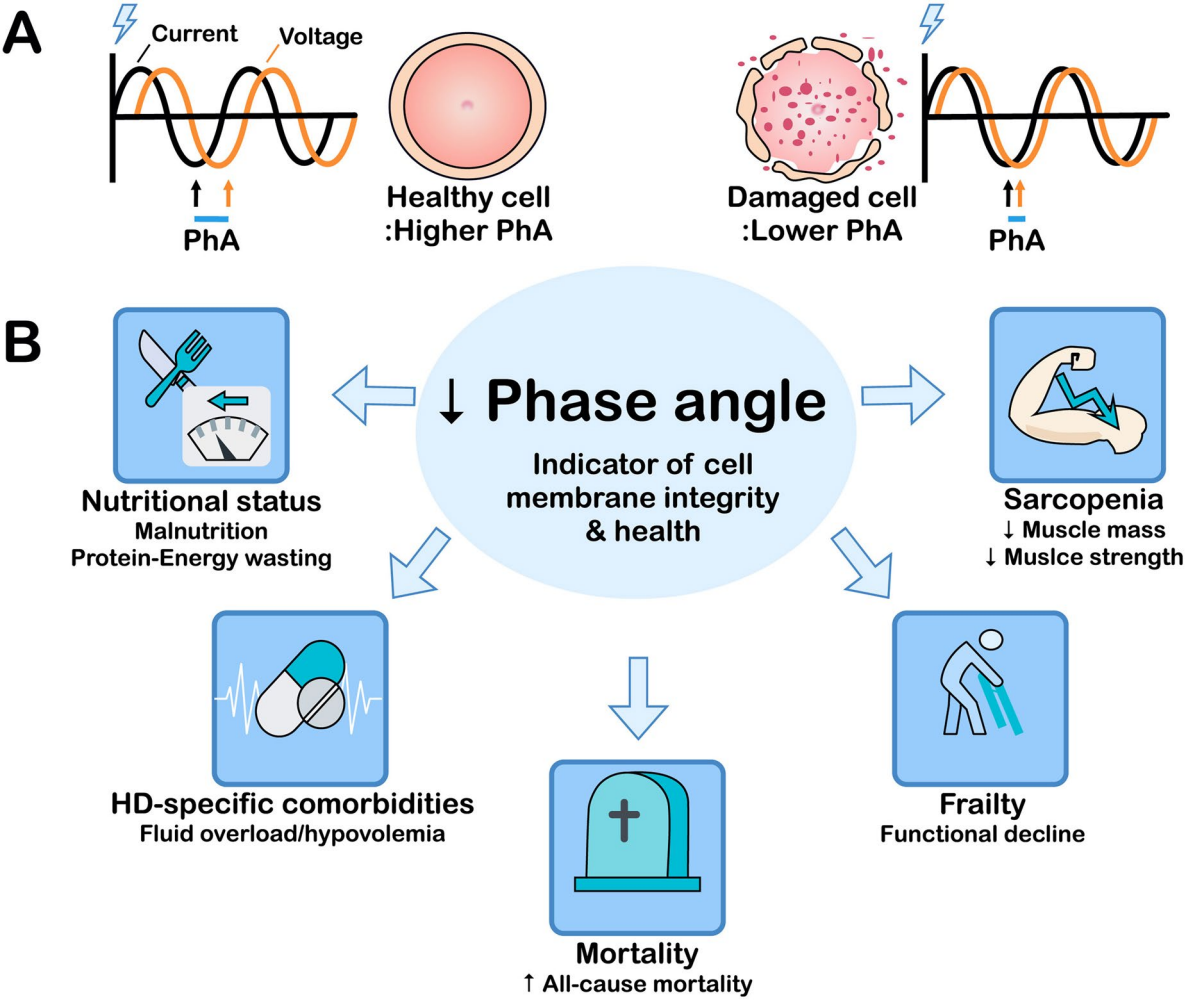
Clinical relevance of the PhA in patients undergoing maintenance hemodialysis. (A) Representative waveforms of applied voltage (black) and measured current (orange) illustrating a larger PhA in healthy cells and a smaller PhA in damaged cells. (B) As an indicator of cell health, a low PhA is related to malnutrition/protein-energy wasting, volume imbalance, and increased risks of sarcopenia, frailty, and mortality. PhA, phase angle.

Although BIA generates various parameters, the direct clinical application of these parameters may be challenging. The PhA has therefore gained attention as a relatively easy-to-use BIA parameter. The PhA is a comprehensive biomarker that integrates information about cellular integrity, nutritional status, and inflammation. As a direct BIA measurement rather than a derived parameter, the PhA offers unique advantages in the clinical assessment of patients undergoing HD. The PhA is calculated from R and Xc, reflecting the phase shift between the voltage and current. Higher PhA values indicate intact cell membranes and normal cellular activity, whereas lower PhA values indicate membrane damage and cell death (Figure 5(A)); the PhA thus serves as a useful surrogate for cell health. In patients undergoing HD, the evaluation of cellular integrity by the PhA contributes to the comprehensive assessment of nutritional status [56] (Figure 5(B)) and also reflects inflammation [57]. During inflammation, Xc decreases due to reduced membrane capacitance, while R also declines owing to increased ECW. Because the reduction in Xc exceeds that of R, the PhA decreases. A previous study demonstrated dynamic relationships between longitudinal changes in PhA and inflammatory biomarkers such as interleukin 6 (IL-6), with each 1 pg/mL increase in IL-6 associated with decreased PhA levels over time [58]. The PhA is useful for assessing the overall health of patients, especially those with chronic diseases, and is an important prognostic indicator [9,59]. Thus, the PhA is useful for predicting the prognosis of and formulating treatment strategies for patients undergoing maintenance HD. Monitoring the PhA can also help evaluate the effectiveness of nutritional interventions and therapeutic strategies.

### Mortality prediction

Over the past two decades, numerous studies have demonstrated that lower PhA values are significantly correlated with increased all-cause mortality in patients undergoing HD [26,57]. Shin et al. found that a PhA below approximately 4.5° was associated with a persistently higher risk of death after adjusting for age, sex, and comorbidities (HR 0.56; 95% CI 0.33–0.97) [60]. Another retrospective analysis also identified 4.5° as an optimal cutoff value for the prediction of mortality, with patients below this threshold experiencing a 2.6-fold increased risk compared with those at or above 4.5° [61].

Notably, meta-analyses have indicated that this cutoff varies slightly among studies, ranging from 4.0° to 5.0° depending on factors such as ethnicity, body mass index, and sex, with one study reporting an interval of 4.54°–5.25° [62]. Although a PhA of 4.5° has been widely used as a reference point, this threshold may not be universally applicable across all populations and clinical settings. The optimal cutoff value appears to be population-specific and may require validation in different ethnic groups and healthcare systems (Table 2).

**Table 2.** PhA Reference values in patients undergoing HD as reported by global studies.

Study	Country	n	Male sex (%)	Age (years)	Mean PhA (°)	Mortality/poor prognosis cutoff (°)	Devices
Maggiore (1996) [63]	Italy	131	50.4	63 ± 14	Pre-HD: 4.2 ± 1.0 Post-HD: 5.1 ± 1.3	M: <4.5 F: <4.2	BIA 101 (Akern)
Chertow (1997) [90]	USA	3,009	52.8	61 ± 16	4.8 ± 1.8	<4.0	BIA Quantum
Abad (2011) [91]	Spain	164	60.4	61 ± 15	7.8 ± 1.2	<8	Bioscan multifrequency analyzer
Beberashvili (2014) [58]	Israel	91	69.2	64 ± 12	5.1 ± 0.96	ΔPhA < −0.45 over 2 years	Nutriguard-M
Beberashvili (2014) [57]	Israel	250	63.2	69 ± 14	4.7 ± 1.3	–	Nutriguard-M
Rodrigues (2014) [92]	Portugal	181	54.7	66 ± 12	5.6 ± 1.2	≤4.9	BIA Quantum
Segall (2014) [93]	Romania	149	55.0	55 ± 14	6.73 ± 4.85	<5.58	BIA 101 (Akern)
Shin (2017) [60]	Korea	142	52.8	64 ± 13	4.6 ± 1.0	<4.5	InBody S10
Saitoh (2020) [94]	Japan	116	64.7	64 ± 12	4.3 ± 1.1°	–	Seca mBCA 515
Lim (2021) [95]	Malaysia	152	53.3	59 (50–66)	M: 4.62 ± 0.82 F: 3.92 ± 0.88	M: <4.26 F: <3.30	BIA Quantum
Ding (2022) [65]	China	346	61.6	58 ± 14	Sarcopenia: 4.06 ± 0.63 Non-sarcopenia: 5.15 ± 0.95	M: <4.67 F: <4.6	Seca mBCA 515
Kang (2022) [64]	Korea	83	51.8	58 ± 13	–	<3.89	InBody S10
Zeni (2023) [96]	Brazil	82	51.2	53 ± 17	Pre-HD: 5.0 ± 1.1 Post-HD: 6.0 ± 1.5	<5.5	BIA 450^™^ (Biodynamics, USA)
Wang (2024) [97]	China	161	68.3	–	–	<5.0 (PEW risk, not mortality per se)	InBody S10
Xu (2024) [61]	China	114	58.8	Control: 54 ± 14 Death: 64 ± 14	Control: 5.90 ± 1.35 Death: 4.72 ± 1.85	<4.5	InBody S10

PhA, phase angle; HD, hemodialysis; M, male; F, female; PEW, protein-energy wasting; ΔPhA, change in phase angle over time.

The relationship between a low PhA and increased mortality appears to involve the interconnection of multiple pathways, including cellular dysfunction, chronic inflammation, and nutritional deterioration [59]. Low PhA values reflect compromised cell membrane integrity, reduced cellular metabolic capacity, and increased systemic inflammation, indicative of an increased risk of mortality.

The prognostic importance of the PhA is also supported by longitudinal data. In a two-year prospective study of 250 patients undergoing maintenance HD, each 1° increase in the baseline PhA was associated with a 37% reduction in all-cause mortality (HR 0.63; 95% CI 0.48–0.81) and a similar decrease in cardiovascular mortality (HR 0.64) [57]. These associations were somewhat attenuated following adjustment for the malnutrition–inflammation score, implying that the PhA reflects nutritional and inflammatory status.

Large-scale research further supports these findings. A systematic review of 42 dialysis cohorts involving approximately 60,000 patients reported that a 1° decrease in the PhA was linked to a 74% increase in mortality (HR 1.74) [26]. A 2021 meta-analysis of 55 studies involving >100,000 patients undergoing dialysis showed that each 1° increase in the PhA corresponded to a 32% decrease in all-cause mortality (HR 0.676) and significantly fewer cardiovascular events (HR ∼0.74) [27]. Consequently, the PhA has emerged as a robust independent predictor of all-cause and cardiovascular mortality.

### Nutritional status

The PhA is closely associated with nutritional status, especially in patients undergoing HD. In a study of 131 patients undergoing HD, the PhA was negatively correlated with age (r=-0.42) and positively correlated with weight (*r* = 0.25), mid-arm circumference (*r* = 0.29), mid-arm muscle circumference (*r* = 0.36), normalized protein catabolic rate (*r* = 0.22), and serum albumin levels (*r* = 0.46). Higher PhA values indicate better nutritional status and were associated with improved survival, confirming the prognostic value of the PhA [63].

A separate study of 103 patients undergoing HD found that PhA values decreased as the number of GLIM criteria met increased, suggesting that severe malnutrition was correlated with lower PhA values; the PhA was a highly accurate predictor of malnutrition (area under the curve 0.748) [51].

### Sarcopenia prediction

Sarcopenia is defined as decreased muscle mass combined with impaired muscle function. Appendicular skeletal muscle mass can be determined by BIA. In addition, the PhA has emerged as a valuable indicator of muscle function.

A study of 250 patients undergoing HD found that PhA values were positively correlated with handgrip strength in both men (*r* = 0.380) and women (*r* = 0.356) [57], suggesting that the PhA may serve as a reliable indicator of muscle health. Another study of 83 patients undergoing HD found that the PhA was positively correlated with handgrip strength (*r* = 0.485; *p* < 0.001) and muscle mass indexed by body surface area (*r* = 0.517; *p* < 0.001), and to a slightly lesser extent with the Subjective Global Assessment, gait speed, and the 30-s sit-to-stand test [64]. These correlations persisted following multivariate analysis, identifying the PhA as an independent surrogate of muscle mass and function.

The PhA has also been shown to support the diagnosis of sarcopenia. A study of 346 patients undergoing HD demonstrated that the PhA had good sensitivity and specificity for identifying sarcopenia according to the Asian Working Group for Sarcopenia criteria, with an area under the curve of 0.823 (*p* < 0.001) in men and 0.830 (*p* < 0.001) in women [65]. A meta-analysis of 18 studies involving 6,184 participants found that the PhA was effective in screening for sarcopenia, with an area under the curve of 0.81, sensitivity of 80%, and specificity of 70%. The 95% CI for the optimal PhA cutoff value was 4.54–5.25. Factors such as ethnicity, body mass index, health status, and diagnostic criteria may influence the ability of the PhA to predict sarcopenia [62].

Muscle quality is a relatively new concept that encompasses muscle size, function, and composition. The European Working Group on Sarcopenia in Older People includes “micro- and macroscopic changes in muscle structure and composition, as well as muscle function delivered per unit of muscle mass” in its definition of muscle quality [66]. Thus, even if two individuals have the same muscle mass, their muscle quality may differ. The PhA can be used as an indicator of muscle quality [67]. In a study of 1,419 young adults and community-dwelling older adults, PhA values were independently correlated with muscle quality, as defined by the ratio of handgrip strength to muscle mass [68].

A longitudinal study of 149 participants showed a linear correlation between PhA values and the skeletal muscle index, handgrip strength, and gait speed, as well as criteria for muscle mass and strength reduction [69]. Participants with a PhA increase of ≥0.2° in a year experienced an increase in the appendicular skeletal muscle mass index, while those with a PhA decrease of >0.2° in a year showed a decline in handgrip strength (*p* = 0.054).

Recent studies evaluating the effects of nutritional and exercise interventions on longitudinal PhA changes have shown promising results [49,70,71], supporting the potential value of the PhA as a surrogate marker in both clinical trials and practice.

### Frailty prediction

Frailty, which encompasses sarcopenia, overall physical weakness, and functional decline, is an important prognostic factor in patients undergoing HD. In a study of 244 patients undergoing maintenance HD, the PhA was effective for predicting pre-frailty, with optimal cutoff values of 6.05° for men (area under the curve 0.919; sensitivity 94.5%; specificity 91.3%) and 5.25° for women (area under the curve 0.870; sensitivity 70.5%; specificity 90.6%) [72]. The addition of a cutoff time of 12.95 s for the five-times sit-to-stand test further increased the ability to predict frailty, indicating that combining the PhA with functional assessments provides a more accurate evaluation.

Given these findings, routinely integrating the PhA into nutritional and prognostic evaluations of patients undergoing HD may facilitate the early identification of those at high risk. In addition, PhA measurement can contribute to nutritional status assessment, survival prediction, determination of infection risk, and guiding anemia treatment, ultimately helping to optimize patient management in clinical practice.

## Limitations and pitfalls of BIA and the PhA

While BIA and the PhA are practical tools for assessing body composition and nutritional status in patients undergoing HD, there are clear limitations and challenges in their clinical interpretation and application.

The fundamental limitation of BIA stems from inter-device variability and operational deviation despite standardized measurement protocols. As manufacturers use different frequencies and proprietary algorithms, parameters derived from specific devices cannot be used interchangeably [73]. Most prediction equations have been developed based on healthy populations, which can lead to accuracy issues when applied to patients with conditions such as chronic kidney disease, which is characterized by fluid imbalance and muscle wasting [74,75]. Deviations among devices also exist. MF-BIA devices have been reported to systematically overestimate body water content compared to BIS devices [76,77]. Furthermore, whole-body BIA has physiological limitations leading to the underestimation of trunk volume, which contributes less resistance compared to limbs. Although segmental BIA partially compensates for this, differences in measurement approaches remain a factor in result interpretation [78].

A major factor limiting the clinical utility of BIA and the PhA is the absence of standardized cutoff values. As the PhA varies significantly differences according to race, sex, and age [79,80], applying a single cutoff value to all patient populations risks misdiagnosis [81]. The complex pathophysiology of patients with HD further complicates BIA interpretation. Fluid overload increases extracellular fluid, lowering electrical resistance and decreasing the PhA, which can mask actual muscle loss [26]. Measurement errors particularly increase when ultrafiltration volume exceeds 2.5 L [82], and measurements taken on arms with arteriovenous fistulas can be affected [83]. Additionally, dialysate sodium concentration can influence post-dialysis fluid redistribution, potentially affecting BIA accuracy in ways that remain incompletely understood [84].

BIA was traditionally contraindicated in patients with cardiac implantable electronic devices, such as pacemakers or implantable cardioverter-defibrillators, due to theoretical risks of electromagnetic interference. However, recent studies have reported that the microcurrent from BIA did not affect implantable electronic device function [85–87], supporting the safe use of BIA in this patient population. Nevertheless, caution and consultation with electrophysiology specialists remain still recommended.

## Future directions

To fully realize the clinical potential of BIA and the PhA, future research should focus on the following priorities. First, large-scale randomized controlled trials are needed to verify whether BIA-guided interventions such as dry weight control and nutritional interventions effectively improve clinical outcomes such as mortality and hospitalization rates. Second, development prediction equations specific for patients with chronic kidney disease undergoing HD, and validated against standard reference methods such as DEXA, is necessary. Third, sophisticated prognostic prediction models integrating BIA data with various clinical variables should be developed using artificial intelligence technologies. Finally, clinical value should be maximized through multi-modal assessment approaches that track longitudinal changes within individuals beyond single time-point measurements and integrate these with functional assessments such as muscle strength testing.

Emerging modalities, such as bioelectrical impedance-based imaging techniques, hold promise for real-time, regional monitoring in clinical settings [88]. Portable, inexpensive devices validated against gold standard assessments could improve accessibility for routine use in satellite dialysis units and homes, particularly when coupled with cloud-based analytics [89]. Integrating BIA-derived indices with traditional nutritional biomarkers such as albumin and prealbumin and inflammatory biomarkers such as IL-6 and C-reactive protein may yield composite prediction models that enhance prognostic risk stratification. Specific cutoff values for BIA parameters including the PhA should be established for different patient populations and devices. Finally, consensus on measurement protocols covering electrode placement, patient positioning, and timing relative to dialysis is essential for inter-center comparability and potential regulatory endorsement.

## Conclusion

BIA is a useful tool for assessing body composition and predicting prognosis in patients undergoing maintenance HD, although limitations such as accuracy in patients with fluid overload, inter-device variability, and lack of standardization remain. Until accuracy improves and specific cutoff values are developed for different patient populations and devices, BIA, and in particular the PhA, should be carefully utilized as a part of comprehensive multi-modal assessments including clinical evaluations, laboratory data, and functional assessments. Future research is required to demonstrate the clinical effectiveness of BIA-guided interventions and describe how technical limitations can be overcome. This would allow BIA to evolve beyond a promising practical tool to provide precision medicine and substantially improve patient outcomes.

## Data Availability

Data sharing is not applicable to this article as no new data were created or analyzed in this study.
